# Targeting the tumor vasculature with TEM8-specific T cells

**DOI:** 10.1186/2051-1426-2-S3-P42

**Published:** 2014-11-06

**Authors:** LaTerrica C Williams, Sunitha Kakarla, Simone Krebs, Thuy Phung, David Rowley, Stephen Gottschalk

**Affiliations:** 1 Baylor College of Medicine, Houston, TX, United States; 2 Baylor College of Medicine/Texas Childrens Hospital/Center for Cell and Gene Therapy, Houston, TX, United States

## Background

T cell immunotherapy with genetically modified T cells expressing chimeric antigen receptors (CARs) has shown promise in preclinical models as well as early clinical studies. However, patients with solid tumors often do not respond as well as patients with hematological malignancies. This lack of efficacy for solid tumors is most likely due to several factors including a) emergence of immune escape mutants, and b) inability of tumor-specific T cells to recognize and destroy the vascular bed of solid tumors, which is critical for their malignant growth. The aim of this project is to generate CARs specific for tumor endothelial marker (TEM) 8, and evaluate their anti-vasculature and anti-tumor activity in preclinical tumor models.

## Methods/results

We generated a retroviral encoding a TEM8-specific CAR consisting of the TEM8-specific single chain variable fragment AF344, a hinge/transmembrane domain, and a CD28.41BB.z endodomain. CD3/CD28-activated T cells were transduced with RD114-pseudotyped retroviral particles to generate TEM8-specific T cells and CAR expression was confirmed by FACS analysis. To evaluate the functionality of TEM8-specific T cells we used TEM8-negative cell lines (U373, A549, LM7, 293T) and 293T cells that were genetically modified to either express human TEM8 (293T.hTEM8) or murine TEM8 (293T.mTEM8). TEM8-specific T cells recognized target cells in an antigen-dependent fashion as judged by their ability to secrete pro-inflammatory cytokines (IFN-g and IL-2) in coculture assays, and kill TEM8-positive target cells. Importantly, TEM8-specific T cells readily recognized mTEM8-positive target cells, which will allow us to evaluate the safety and efficacy of TEM8-specific T cells in xenograft and immune competent murine tumor models.

## Conclusion

We have constructed a TEM8-specific CAR and have shown that T cells expressing this CAR recognize and kill hTEM8- or mTEM8-positive target cells. Animal studies are in progress to determine their safety and efficacy. Targeting the tumor vasculature with TEM8-specific T cells may improve current T cell immunotherapies for solid tumors.

